# Harmonization of slice thickness through resampling improves comparability of MRI-derived neonatal brain volumes

**DOI:** 10.3389/fradi.2026.1868602

**Published:** 2026-06-29

**Authors:** Anouk S. Verschuur, Martijn F. Boomsma, Alexander Leemans, Ingrid M. Nijholt, Leonora Hendson, Selma Low, Amy Metcalfe, Donna M. Slater, Chantal M. W. Tax, Gerda Meijler, Lara M. Leijser

**Affiliations:** 1 Department of Radiology, Isala Hospital, Zwolle, Netherlands; 2 Image Sciences Institute, University Medical Center Utrecht, Utrecht, Netherlands; 3 Department of Pediatrics, Section of Newborn Critical Care, University of Calgary, Calgary, AB, Canada; 4 Division of Imaging and Oncology, University Medical Center Utrecht, Utrecht, Netherlands; 5 Department of Obstetrics and Gynecology, University of Calgary, Calgary, AB, Canada; 6 Department of Physiology and Pharmacology, University of Calgary, Calgary, AB, Canada; 7 Departments of Community Health Sciences and Medicine, University of Calgary, Calgary AB, Canada; 8 CUBRIC, School of Physics and Astronomy, Cardiff University, Cardiff, United Kingdom; 9 Department of Pediatrics, Division of Neonatology, Leiden University Medical Center, Leiden, Netherlands

**Keywords:** brain volume, MRI, neonate, segmentation, slice thickness

## Abstract

**Purpose:**

To assess reliability and agreement of brain volume measurements from neonatal T2-weighted magnetic resonance imaging (MRI) scans acquired with different slice thickness settings (i.e., 2 mm, 3 mm and 3 mm resampled).

**Methods:**

We used term-equivalent (38–45 weeks postmenstrual age) T2-weighted MRI scans with slice thicknesses of 2 mm and 3 mm from a subset of 40 preterm and term infants (27–40 weeks' gestation) from two existing neonatal imaging cohorts. Resampling of the 2 mm scans to a 3 mm grid provided a third set of scans, referred to as “3 mm resampled”. Automated brain segmentation provided intracranial volume, total brain volume, and volumes for eight brain structures. Reliability and agreement of volume measurements were assessed using intraclass correlation coefficients (ICC) and Bland-Altman plots, respectively.

**Results:**

For all volumes, inter-scan reliability was higher for 3 mm vs. 3 mm resampled scans (mean observer ICC >0.92) compared to 2 mm vs. 3 mm scans (mean observer ICC >0.87). Bland-Altman plots revealed smaller mean volume differences between 3 mm and 3 mm resampled scans than between 2 mm and 3 mm scans. However, a proportional bias was found for deep gray matter volume for 3 mm vs. 3 mm resampled scans.

**Conclusion:**

Slice thickness influences brain volume measurements from neonatal MRI. Harmonization of imaging data acquired with different settings through image resampling improves reliability and agreement of brain volume measurements.

## Introduction

Over the past three decades, brain volumes in neonatal populations have been studied to better understand early brain growth in relation to adverse perinatal outcomes such as preterm birth, asphyxia, brain injury and congenital heart disease (1–4). Altered neonatal brain volume may result from adverse perinatal exposures (5) and is associated with long-term neurodevelopmental outcomes (6, 7). A recent meta-analysis aimed to describe reference ranges for cerebral and cerebellar volumes from MRI at term-equivalent age (TEA) in very preterm and very low birthweight infants (1). However, a major methodological limitation in comparing brain volumes across studies is heterogeneity in MR image acquisition and processing (e.g., voxel size, echo time, repetition time, and segmentation algorithm) (1). This heterogeneity across studies can limit reproducibility and generalizability of results and potentially introduce bias when comparing data from different studies.

The subtle yet potentially clinically relevant differences in brain volumes across the gestational age spectrum necessitate large, combined datasets obtained in preterm infants to achieve meaningful results. However, combining imaging data from different cohorts often relies on data acquired using different MR systems and settings, particularly slice thickness and voxel size. Seemingly minor differences, such as voxel size, can affect brain volumetric measurements and potentially under- or overestimate subtle brain volume differences in the preterm population.

The current study focuses on the effects on brain volume measurements of varying slice thickness and data harmonization through resampling, taking advantage of an existing dataset of neonates with two T2-weighted scans acquired with differing slice thicknesses but otherwise identical settings. Our specific aim was to investigate the reliability and agreement of brain volume measurements derived from T2-weighted images with 2 mm, 3 mm, and 3 mm resampled slice thicknesses in neonates.

## Methods

**Figure 1:**
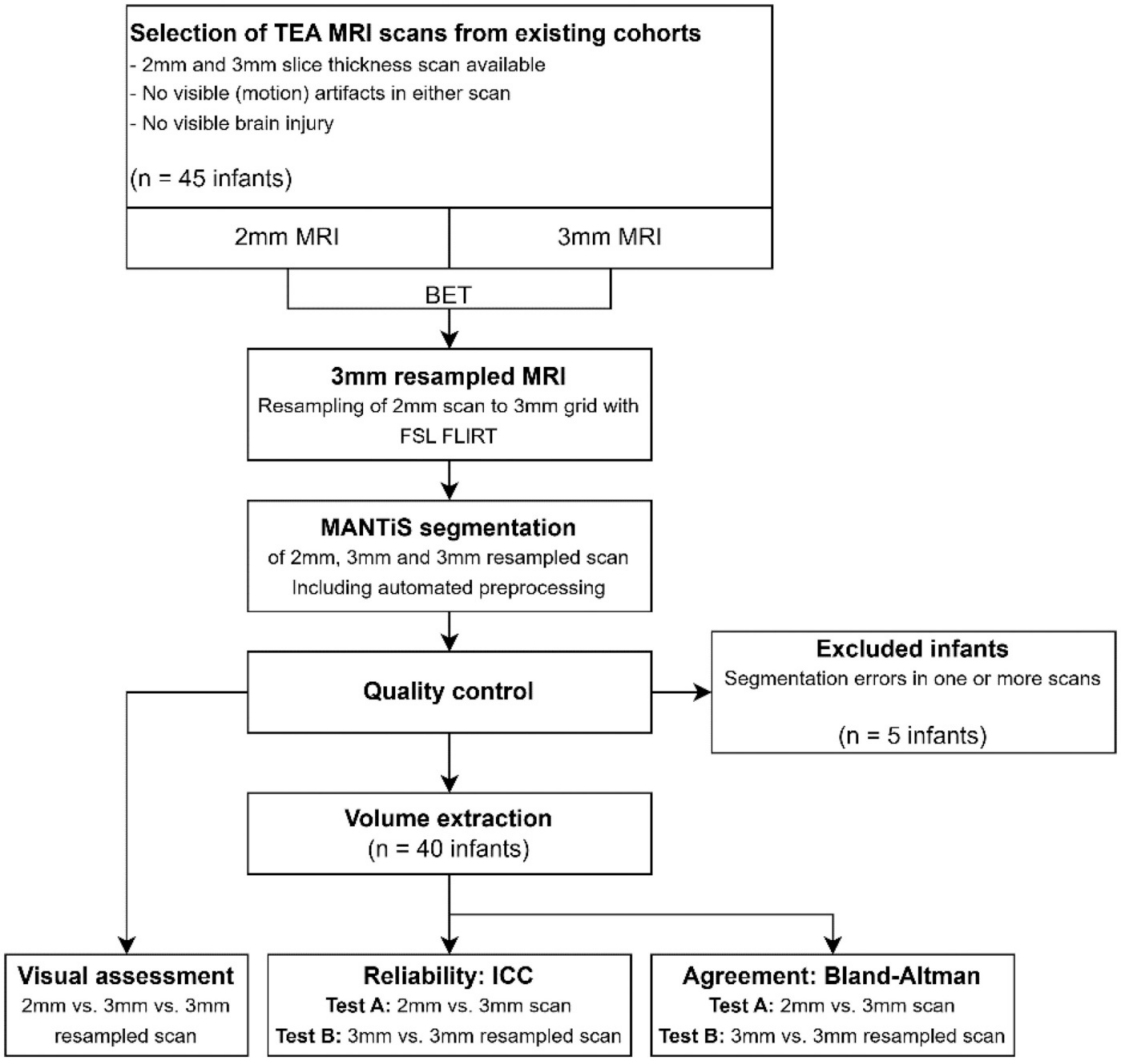
Workflow diagram. TEA, term equivalent age; BET, brain extraction, ICC, intraclass correlation coefficient.

This study retrospectively used a randomly selected subset of available imaging data from two prospective Canadian preterm cohorts with ethics approval from the Research Ethics Board (REB) at the University of Calgary, 1) the “brain imaging in moderate-late preterm infants (BIMP) study” (REB19-1194), including moderate-late preterm infants imaged between November 2020 and March 2023, and 2) the “P3 brain health study” (REB21-1446), including preterm and full-term infants imaged between March 2022 and March 2024. All included infants underwent brain MRI around TEA (38–45 weeks postmenstrual age) on a research-dedicated 3 Tesla GE MR750W system at the Alberta Children's Hospital, Calgary. Infants were scanned non-sedated using the feed-and-sleep technique (8) a vacuum mattress and head cushioning to reduce motion artifacts from movement and waking. Exclusion criteria for both study cohorts were congenital malformations or infections of the central nervous system, chromosomal disorders and inborn errors of metabolism. Written informed consent was obtained from the parents. For the purpose of this study, 45 infants were selected in whom two T2-weighted scans were obtained, i.e., one with slice thickness of 2 mm (0 mm gap) and one with slice thickness of 3 mm (0.4 mm gap) (see scan parameters in Table 1 and workflow in Figure 1). For the selection of this sub-cohort, strict criteria were applied to eliminate the impact of imaging artifacts on volumetric measurements; infants were excluded if any (motion) artifacts were detected on visual inspection (by ASV) in either of the two T2-weighted MRI scans. None of the infants included had visible brain injury (assessed by LML and GM).

**Table 1:** Scan parameters for the 2 mm and 3 mm T2-weighted MRI.

Parameter	2 mm	3 mm
Acquisition time	3:14 min	1:37 min
Acquisition plane	Axial	Axial
Pixel size	0.375 × 0.375 mm	0.375 × 0.375 mm
Slice thickness	2 mm	3 mm
Gap	0 mm	0.4 mm
Number of slices	51	30
TE	120 ms	120 ms
TR	4,400 ms	4,400 ms
Flip angle	111°	111°
Echo train length	18	18
Acquisition matrix	320 × 320	320 × 320
Reconstruction matrix	512 × 512	512 × 512
FOV	192 mm	192 mm
% FOV	100	100
SNR	12.9	18.9

TE, echo time; TR, repetition time; FOV, field of view; SNR, signal to noise ratio.

### Data processing and visual assessment

All axial T2-weighted scans were skull stripped using the brain extraction tool from FMRIB Software Library (FSL, version 6.0.3; threshold 0.5) (9, 10). Extracerebral tissue was manually removed if still present during visual quality control. In addition to the 2 mm and 3 mm scans, a third scan was created for each infant to evaluate the effect of harmonization through resampling; 2 mm scans were down-sampled to the grid of the 3 mm scan (FSL FLIRT; settings: trilinear interpolation with affine registration), generating a “3 mm resampled” scan (i.e., with 0.4 mm gap) (11, 12). Only down-sampling to a thicker slice resolution was performed, as up-sampling would introduce interpolation effects and therefore potentially generate artificial information not present in the original data. Signal-to-noise ratio (SNR) differences between scan types (i.e., 2 mm, 3 mm and 3 mm resampled) were assessed by measuring mean and standard deviation (SD) of voxel intensities with a fixed-size region of interest placed in homogeneous frontal white matter (WM). To assess volumetric differences between 2 mm, 3 mm and 3 mm resampled scans, brain segmentation was performed from all sets of three T2-weighted scans per infant, using an adapted version of the morphologically adaptive neonatal tissue segmentation toolbox (MANTiS) (13, 14) A total of 10 volumes per scan were calculated, including intracranial volume (ICV), total tissue volume, WM, cerebrospinal fluid (CSF), cortical gray matter (cGM), deep gray matter, hippocampus, amygdala, brainstem and cerebellum. Visual quality control of images included evaluation for noise (graininess of the image) and partial volume effects (tissue edge demarcation), and evaluation of segmentations (superimposed on the T2-weighted scan) for over- or under-segmentation, or incorrect labeling. Scans with segmentation errors were excluded.

### Statistical analyses

Reliability and agreement of brain volumes derived from 2 mm vs. 3 mm scans (test A), and from 3 mm vs. 3 mm resampled scans (test B) were calculated. Reliability of volumetric measurements was assessed using the intraclass correlation coefficient (ICC). ICC values were calculated as described by De Vet et al., with categories adopted from Koo et al. as poor (<0.5), moderate (0.5–0.75), good (0.75–0.9) and excellent (>0.9) reliability (15, 16). In short, mean square error (EMS; $\sigma_{\mathrm{error}}^{2}$; variability within groups), between-subject mean square (BMS; variability between infants), and observation mean square (OMS; variability between scan type) were acquired with ANOVA. $\sigma_{\mathrm{infant}}^{2}$ [Equation (1); the isolated variability between infants] and $\sigma_{\mathrm{observation}}^{2}$ [Equation (2); the isolated variability between observations/scan types] were calculated:

$$\begin{array}{c} \sigma_{\mathrm{infant}}^{2}=\frac{BMS-EMS}{k} \end{array}$$ (1)

$$\begin{array}{c} \sigma_{\mathrm{observation}}^{2}=\frac{OMS-EMS}{n} \end{array}$$ (2)

With “*n*” the number of infants and “k” the number of observations. Subsequently, ICC agreement [Equation (3)] and observation ICC agreement [Equation (4)] were calculated as follows:

$$\begin{array}{c} ICC\;\mathrm{agreement}=\;\frac{\sigma_{\mathrm{infant}}^{2}}{\sigma_{\mathrm{infant}}^{2}+\sigma_{\mathrm{observation}}^{2}+\sigma_{\mathrm{error}}^{2}}\; \end{array}$$ (3)

$$\begin{array}{c} \mathrm{observation}\;ICC=\;\frac{\sigma_{\mathrm{infant}}^{2}}{\sigma_{\mathrm{infant}}^{2}+\frac{\left(\sigma_{\mathrm{observation}}^{2}+\sigma_{\mathrm{error}}^{2}\right)}{k}}\; \end{array}$$ (4)

Bland-Altman plots assessed agreement between measurements, including potential systematic and proportional bias (defined as volume deviations proportional to the mean volume). Bland-Altman plots are visualized with limits of agreement (mean ± 1.96*SD) representing both the systematic error (mean) and the random error (SD). Systematic bias was assessed using a one sample *T*-test on the volume difference, and proportional bias was evaluated using a linear regression of the volume difference against the mean volume. Significance was set at *p* < 0.05.

For all analyses, a pre-defined 5% deviation from the average brain volume was considered an acceptable level of agreement. The 5% threshold was informed by previous work comparing brain volumes between neonatal cohorts, in which all statistically significant differences exceeded 5% of the mean volume (17). Statistical analyses were executed with IBM SPSS statistics (version 28.0.1.1, IBM SPSS Statistics for Windows, IBM Corp.). Additional calculations were done, and graphs were generated in Python (version 3.8.5, Python Software Foundation, python.org).

## Results

In five of the 45 infants, segmentation failed for at least one of the three MRI scans (2 mm, 3 mm or 3 mm resample). As a complete dataset of three scans was required for within-subject comparisons, all scans from the infants with one or more failed segmentations were excluded from the analyses; 15 out of the 135 scans total were excluded. The included 40 infants [36 preterm (90%)], with 3 scan types each (totaling 120 MRI scans), had a mean [SD] gestational age at birth of 34.8 [2.7] weeks, postmenstrual age at scan of 41.7 [1.6] weeks. 22 preterm infants were male (61%), while 2 full term infants were male (50%).

### Visual assessment

**Figure 2:**
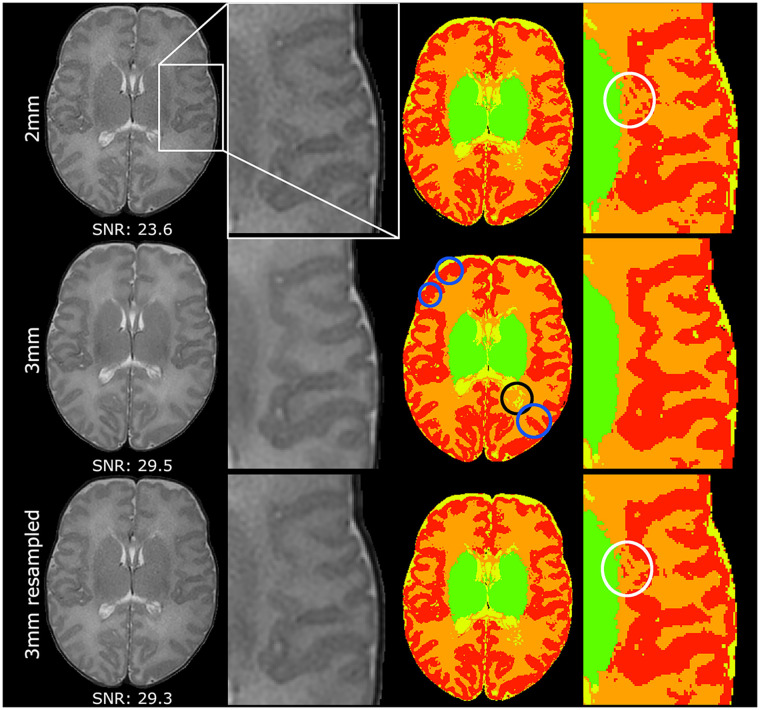
Axial views [with signal-to-noise ratio (SNR)] and zoomed images of the 2 mm, 3 mm and 3 mm resampled scans (left two columns) with their corresponding segmentation results (right two columns). Noise and less sharply demarcated cGM-WM edges, respectively, are visible in the 2 mm and 3 mm scans. Segmentation is visually affected by the noise (white circles) and partial volume effects (blue circles). Of note: The apparent CSF over-segmentation in the 3 mm image (black circle) results from the algorithm misclassifying hyperintense WM, not from noise in the image.

T2-weighted scan quality was affected by the slice thickness setting (MR images in Figure 2). Visually, the 2 mm scans contained the most noise, followed by the 3 mm resampled scan, with the 3 mm scan containing the least noise, consistent with the SNR measurements (see values in Figure 2). The WM-cGM edge demarcation was sharpest on the 2 mm scan, with demarcation being less sharp on the 3 mm scan and the 3 mm resampled scan; the 3 mm and 3 mm resampled scans showed similar demarcation sharpness.

Variation in tissue segmentation was most prominent in the cGM-WM edge demarcation as compared to other structures (zoomed segmentation images in Figure 2). The cGM thickness was slightly increased in the 3 mm scan compared to the 2 mm scan (blue circles in Figure 2). Additionally, the segmentation edge was less irregular on the 3 mm scan, while the 2 mm and 3 mm resampled scan contained several misplaced areas of cGM segmentation in the WM (white circles in Figure 2).

### Reliability

The overall ICC values for all brain volumes were good to excellent in test A (2 mm vs. 3 mm scans) and excellent in test B (3 mm vs. 3 mm resampled scans) (Table 2). The observation ICC was excellent for all brain volumes in both test A and B, except for the hippocampus in test A. The ICC agreement and observation ICC were consistently higher in test B compared to test A, except for the ICV where ICC values were the same between tests. Harmonization (test B comparison) also resulted in lower observation variability ($\sigma_{\mathrm{observation}}^{2}$) for all volumes compared to test A. Notably, a negative observation variability was found for total tissue, CSF and cerebellum volumes in test B.

**Table 2:** Intraclass correlation coefficient (ICC), including ICC between observations, $\sigma_{\mathrm{infant}}^{2}$, $\sigma_{\mathrm{observation}}^{2}$, $\sigma_{\mathrm{error}}^{2}$ for test A (comparing 2 mm and 3 mm scans) and test B (comparing 3 mm and 3 mm resampled scans).

Volume	Test A					Test B				
	ICC agreement	ICC agreement observation	$\sigma_{\mathrm{infant}}^{2}$	$\sigma_{\mathrm{observation}}^{2}$	$\sigma_{\mathrm{error}}^{2}$	ICC agreement	ICC agreement observation	$\sigma_{\mathrm{infant}}^{2}$	$\sigma_{\mathrm{observation}}^{2}$	$\sigma_{\mathrm{error}}^{2}$
ICV	0.996	0.998	3,131.3	1.82	9.48	0.996	0.998	3,122.1	0.16	11.39
Total tissue	0.996	0.998	1,761.0	4.19	3.45	0.998	0.999	1,702.0	−0.08	3.72
cGM	0.976	0.988	636.8	5.22	10.15	0.984	0.992	656.7	0.43	10.29
WM	0.927	0.962	254.2	11.73	8.33	0.964	0.982	264.8	0.07	9.78
CSF	0.952	0.975	568.5	11.85	16.72	0.970	0.985	577.8	−0.16	18.26
dGM	0.983	0.991	6.10	0.04	0.07	0.988	0.994	5.78	0.01	0.06
Hippocampus	0.769	0.870	0.07	0.01	0.01	0.935	0.967	0.07	0.0001	0.01
Amygdala	0.823	0.903	0.04	0.001	0.01	0.863	0.926	0.04	0.0007	0.01
Cerebellum	0.958	0.978	11.74	0.24	0.27	0.990	0.995	10.79	−0.001	0.11
Brainstem	0.924	0.961	0.409	0.02	0.02	0.937	0.967	0.37	0.0002	0.03

ICV, intracranial volume; cGM, cortical gray matter; WM, white matter; CSF, cerebrospinal fluid; dGM, deep gray matter.

### Agreement

**Figure 3:**
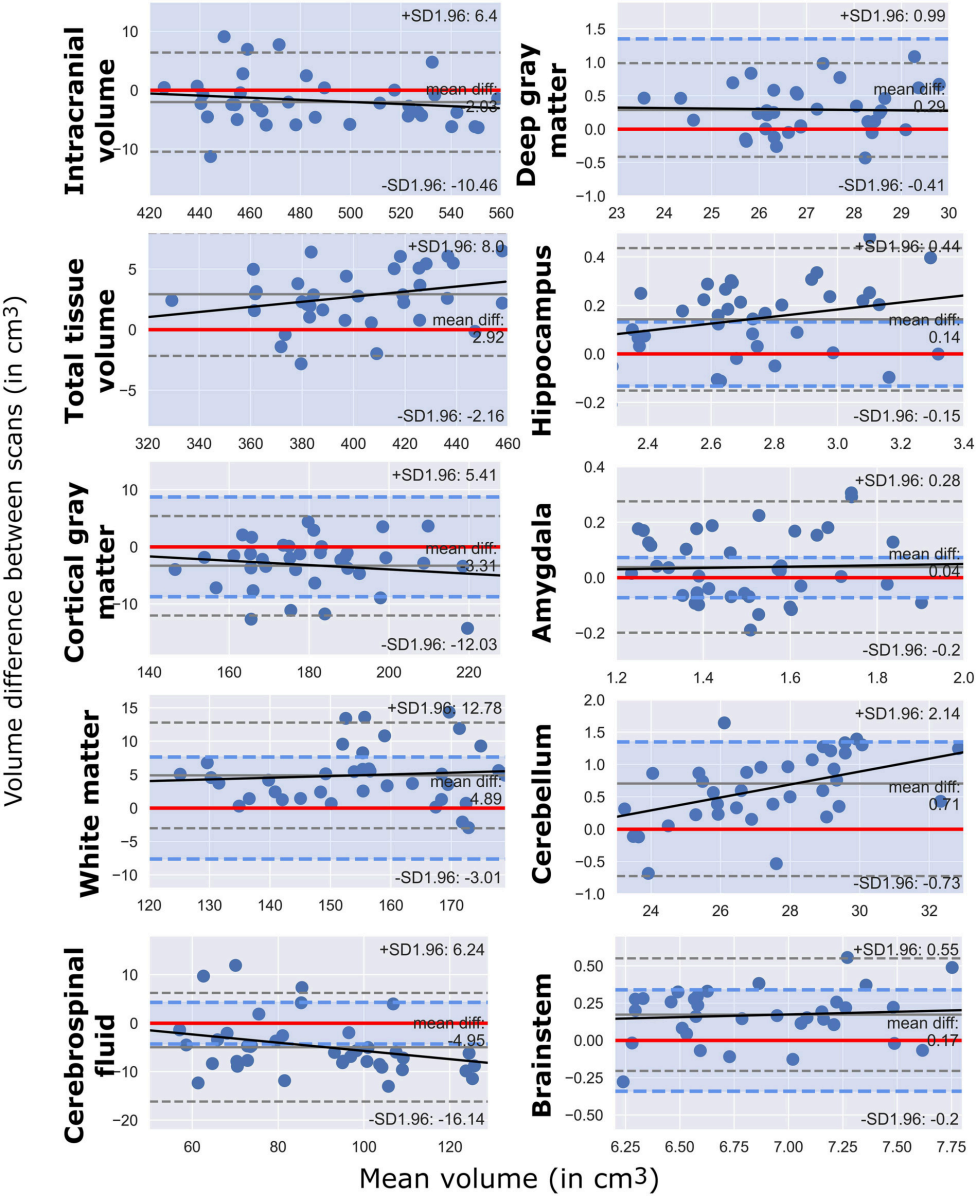
Bland-Altman plots for assessment of systematic and proportional bias for brain volume measurements from 2 mm and 3 mm slice thickness images (test A). X-axes: combined mean volume; y-axes: volume difference; gray line: mean difference; dashed lines: limits of agreement; black line: regression for proportional bias; red: 0-line; blue line with surface: pre-defined 5% acceptance range.

**Figure 4:**
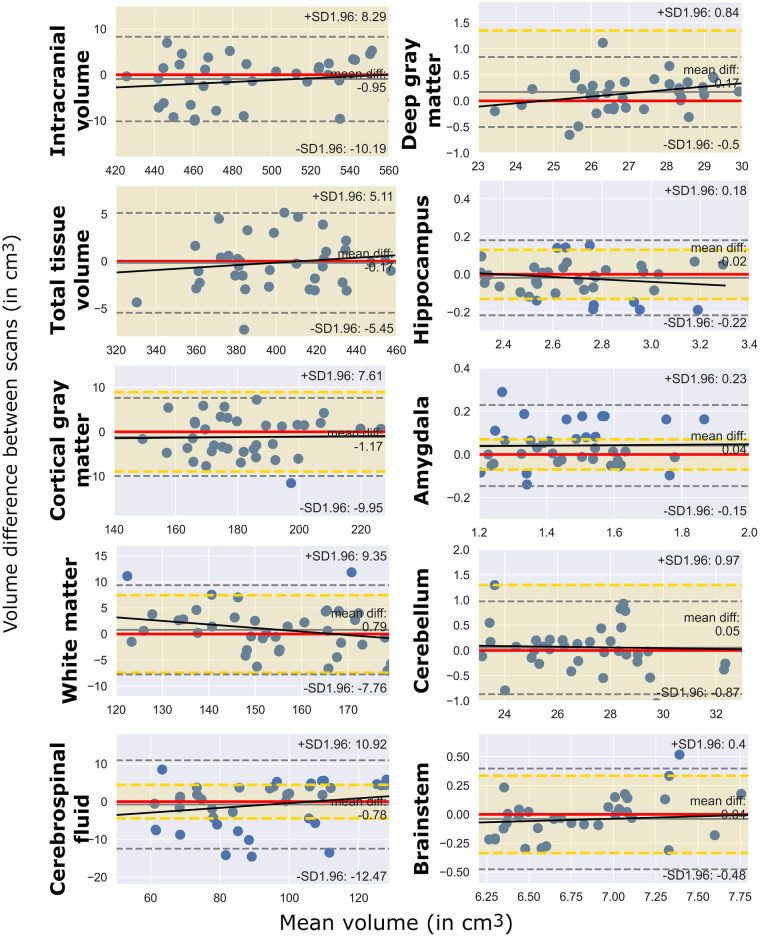
Bland-Altman plots for assessment of systematic and proportional bias for brain volume measurements from 3 mm and 3 mm resampled slice thickness images (test B). X-axes: combined mean volume; y-axes: volume difference; gray line: mean difference; dashed lines: limits of agreement; black line: regression for proportional bias; red: 0-line; yellow line with surface: pre-defined 5% acceptance range.

Bland-Altman plots for test A and B (Figures 3, 4, respectively) show the volume differences plotted against the mean volumes for all tissues. Test B (Figure 4) shows a consistently smaller mean difference [gray line deviating from the 0-line (in red)] compared to test A (Figure 3). Systematic bias (Table 3) observed in test A for all tissue volumes (*p* < 0.005), except amygdala (*p* = 0.056), is decreased (dGM; *p* = 0.004), or no longer observed (remaining volumes; *p* ≥ 0.05) in test B. A new systematic bias was observed for the amygdala (*p* = 0.011) in test B, though the mean difference did not change. Additionally, proportional bias (Table 3) was found for total brain tissue (*p* = 0.034), CSF (*p* = 0.025) and cerebellum (*p* = 0.002) volumes in test A. While these proportional biases for total brain, CSF and cerebellum were no longer observed in test B (*p* = 0.235, *p* = 0.113 and *p* = 0.76, respectively), a proportional bias was found for dGM volumes in test B (*p* = 0.004 vs. *p* = 0.767 in test A). Volumetric differences for test B were more frequently within the 5% range of acceptance (colored surfaces Figures 3, 4) than in test A. In test A, the mean difference for CSF and hippocampus was outside the 5% range, whereas in test B none of the mean differences fell outside the 5% range.

**Table 3:** Statistical analyses accommodating the bland-altman plots. Analyses for systematic and proportional bias are provided for Test A and B.

Test A	Systematic bias				Proportional bias		
	Mean difference	95%-CI low	95%-CI high	*p*-value	Slope	Intercept	*p*-value
ICV	−2.03	−3.42	−0.64	0.005	−0.018	6.99	0.15
Total tissue	2.92	2.08	3.76	<0.001	0.021	−5.68	0.034
cGM	−3.31	−4.75	−1.87	<0.001	−0.038	3.64	0.191
WM	4.89	3.58	6.19	<0.001	0.025	1.02	0.553
CSF	−4.95	−6.80	−3.10	<0.001	−0.085	2.77	0.025
dGM	0.29	0.17	0.40	<0.001	−0.007	0.48	0.767
Hippocampus	0.14	0.09	0.19	<0.001	0.145	−0.25	0.094
Amygdala	0.04	−0.001	0.08	0.056	0.024	0.001	0.81
Cerebellum	0.71	0.47	0.94	<0.001	0.1	−2.11	0.002
Brainstem	0.17	0.11	0.24	<0.001	0.036	−0.08	0.464
Test B							
ICV	−0.95	−2.47	0.58	0.217	0.02	−11.20	0.137
Total tissue	−0.17	−1.04	0.70	0.693	0.013	−5.35	0.235
cGM	−1.17	−2.62	0.28	0.110	0.006	−2.30	0.211
WM	0.79	−0.62	2.21	0.263	−0.068	11.34	0.114
CSF	−0.78	−2.71	1.16	0.422	0.063	−6.71	0.113
dGM	0.17	0.06	0.28	0.004	0.064	−1.58	0.004
Hippocampus	−0.02	−0.05	0.01	0.266	−0.065	0.16	0.339
Amygdala	0.04	0.01	0.07	0.011	0.011	0.03	0.894
Cerebellum	0.05	−0.10	0.21	0.487	−0.007	0.25	0.76
Brainstem	−0.4	−0.11	0.03	0.278	0.041	−0.33	0.486

CI, confidence interval; ICV, intracranial volume; cGM, cortical gray matter; WM, white matter; CSF, cerebrospinal fluid; dGM, deep gray matter.

## Discussion

The aim of this study was to assess the reliability and agreement of brain volume measurements from neonatal T2-weighted MRI scans with different slice thicknesses. Our findings show that slice thickness influences volumetric measurements of neonatal brain structures. Specifically, differences in volumes derived from 2 mm and 3 mm scans resulted in lower reliability and agreement, whereas resampling 2 mm scans to 3 mm improved both reliability and agreement across all evaluated brain volumes. Together, the results highlight the importance of slice thickness harmonization and indicate that resampling improves comparability of brain volume measurements across acquisitions with different resolutions. Data harmonization through resampling may therefore contribute to improved comparability across datasets and support future multi-cohort neonatal MRI research.

Visual inspection of the tissue contrasts of different scan types and the segmentations suggested differences related to slice thickness. Although these differences in segmentation appeared subtle (e.g., for cGM thickness), marginal discrepancies in segmentation boundaries across slices can become more pronounced in whole brain segmentation and result in measurable yet potentially unreliable differences in volumetric estimates.

Reliability analyses using the ICC framework showed consistently higher reliability after resampling. The higher overall ICC and observer ICC, together with the lower observer variance component ($\sigma_{\mathrm{observation}}^{2}$), suggest that the influence of the observer (i.e., slice thicknesses) on the measurements decreased after harmonization. These findings indicate that resampling to a uniform slice thickness of 3 mm reduces measurement variability and improves the consistency of segmentation-derived brain volumes. Notably, three negative observer variance components were obtained in test B. Negative observation variance [Equation (2)] can occur if the variance in volumes is greater between study subjects than the variance introduced by observers. For example, the variance in CSF volumes across infants scanned at different postmenstrual ages was higher than the minimal variance observed between 3 mm and 3 mm resampled slice thicknesses. The negative observation variance supports the assumption of negligible observation variance, suggesting that observer-related variance for total tissue, CSF and cerebellum volumes contributed only minimally to the overall measurement variability in test B.

Agreement analyses using Bland-Altman plots showed a similar pattern. The mean differences between scans were consistently smaller in test B than in test A, indicating improved agreement after resampling. In addition, proportional bias was no longer observed for total tissue, CSF and cerebellum volumes following harmonization of slice thickness. However, dGM showed a newly observed proportional bias in test B, with increasing volume differences between scans at larger mean volumes. The cause of this finding is unclear but it may be related to interpolation effects introduced during resampling. Interpolation can alter boundary voxels through partial volume effects, particularly at the boundaries of structures. As larger structures have more boundary voxels, minimal shifts in the segmentation boundary may result in larger absolute volume differences than those observed for smaller structures, potentially contributing to the observed proportional bias.

Given the limited sample size typical for single-center neonatal MRI studies, multi-center collaborations to achieve large datasets are essential for meaningful results, advancing our understanding of early brain development, and informing neuroprotective strategies. Pooling of neonatal cohorts (18), multi-site recruitment (7, 19, 20), or multi-center comparative-effectiveness studies allow for robust comparisons and increase statistical power. Homogeneity of MRI acquisition settings and systems across pooled cohorts may improve the comparability and generalizability of the study results, whereas heterogeneity of acquisition may introduce bias. A neonatal study that developed and validated a deep learning harmonization tool for cortical thickness measurements found that data harmonization improved the consistency of cGM volume and thickness between cohorts (21). Several studies in adults have also investigated the effect of imaging settings and scan-rescan reliability on brain volume measurements (22, 23). These studies, comparing measurements from scans acquired in adults at 1-hour and 1-week intervals, found within-subject differences in brain volume measurements across different MRI systems (23) and in small brain structures, such as the amygdala (with mean differences of up to 0.19 cm^3^) (22). The impact and clinical relevance of these findings may be even greater in the smaller neonatal brain, where each voxel represents a much larger portion of the total brain tissue than in adult scans. Though, the influence of acquisition differences may vary depending on the study design and outcomes. For example, distributing the recruitment and imaging of a single cohort across multiple sites may be less susceptible to measurement differences than comparing separate cohorts (e.g., preterm and term infants) recruited from different sites, as slight variations in image acquisition are distributed across the cohort rather than between groups. In the latter case, resampling of data and/or harmonization of imaging protocols and volumetric outputs may improve reliability and agreement. Regardless, the use of different cohorts with varying acquisition settings warrants careful consideration of results and conclusions.

When deciding on acquisition settings, particularly slice thickness of T2-weighted MRI for volumetric analysis, the trade-off between signal-to-noise ratio and partial volume effects need to be considered. For example, thicker slices reduce acquisition time and noise, but at the expense of an increase in partial volume effects. As shown in Figure 2, the less clear demarcation of the WM-cGM boundaries in the 3 mm scan compared to the 2 mm scans resulted in thicker cGM segmentation, while the increased noise in the 2 mm scan resulted in areas of WM being mislabeled as cGM, potentially leading to an overestimation of cGM in both scans. Another consideration to further improve image quality for segmentation and quantitative analyses is the use of an isotropic voxel size, as opposed to the anisotropic voxel size of the scans in this study, which reduces through-slice partial volume effects (24).

A key strength of the current study is the focus on neonatal brain MRI. While many investigations into the effects of acquisition parameters and image preprocessing strategies have been performed in the adult population, the impact of such differences on neonatal data remains largely unexplored. Neonatal brains present unique challenges due to smaller size, higher water content, rapidly changing tissue properties and contrasts, and greater susceptibility to motion artifacts. As a result, findings from adult datasets cannot be generalized to this population. By examining how differences in slice thickness and resampling affect volumetric measurements in neonates born across the gestational age spectrum, our study addresses a critical knowledge gap. Understanding the effects of slice thickness, in isolation from the effects of other settings, is essential for improving reproducibility, enabling meaningful comparisons across studies, and ultimately facilitating the integration of neonatal MRI data into larger, multi-site research efforts. A unique dataset with paired 2 mm and 3 mm slice-thickness T2-weighted scans, with other settings (e.g., echo time and repetition time) held consistent, enabled a direct comparison of the effect of slice thickness on segmentation results.

However, some limitations should be considered when interpreting these findings. First, this proof of concept study evaluated a single harmonization scenario, comparing volumetric measurements obtained from two slice thicknesses using one segmentation pipeline and a single harmonization approach based on down-sampling. However, there are many other factors that can affect the segmentation output, such as other acquisition settings (voxel size, echo time, repetition time, slice thicknesses), MR vendor/system, preprocessing strategy, resampling approach, segmentation pipeline and population characteristics. Therefore, our results should not be interpreted as evidence that down-sampling to a 3 mm slice thickness is the optimal strategy for harmonizing neonatal MRI data. Rather, our results demonstrate that resampling improves the comparability of brain volume measurements relative to using images with different slice thicknesses. In the absence of a gold standard for volume measurements, we chose to down-sample the 2 mm data to 3 mm resolution rather than up-sampling the 3 mm scan to 2 mm, as up-sampling would introduce interpolated data that could influence volumetric measurements. Nevertheless, a 2 mm slice thickness may be more accurate for brain volume measurements and also more often used in neonatal imaging. Further research is therefore needed to determine the most appropriate harmonization strategy across a wider range of slice thicknesses, acquisition protocols, resampling approaches, and segmentation methods. Second, the difference in slice gap between the 2 mm and 3 mm scans may have induced tissue and volume under-sampling, enhanced partial volume effects, and reduced spatial resolution in the 3 mm scan. Although a 3 mm and 2 mm slice thickness were compared, the slice spacing in this study is 3.4 mm (3 mm slice with 0.4 mm gap) vs. 2 mm. Finally, the 5% acceptance margin was informed by prior brain volumetric research in moderate-to-late preterm infants in the Dutch cohort of the BIMP-study (17), in which mean differences of statistically significant findings were >5% of the mean volumes. We therefore considered differences below this threshold less likely to affect the interpretation of neonatal brain volumetric estimates or have clinical significance, although alternative acceptance margins could also be justified.

Further studies are needed to improve our understanding of the effects of different acquisition settings on neonatal brain volume measurements, in order to optimize MRI protocols prior to the initiation of multi-cohort studies. Increased awareness of image acquisition settings and processing may help to distinguish true brain volume differences from acquisition and processing-induced volume differences between studies and cohorts, and improve reproducibility and comparability (e.g., for meta-analysis) of neonatal brain volume measurements. Overall, careful consideration of potential biases is warranted when comparing existing volume measurements derived from multiple cohorts. Additionally, attention should be paid to the impact of differences in acquisition, and methods to improve data homogeneity should be applied wherever possible before combining datasets.

## Conclusion

Neonatal brain volume measurements are affected by T2-weighted MRI slice thickness in neonates, potentially affecting the sought-after relationships between brain development and neurodevelopmental outcomes. Data harmonization by scan resampling improves the reliability and agreement of volume measurements and may be the best approach to enable multi-center collaborations for expanded cohorts despite MRI acquisition with different settings. However, future studies should evaluate a broader range of acquisition protocols, resampling approaches, and segmentation methods to establish robust harmonization strategies and ultimately improve the reproducibility and comparability of neonatal MRI volumetric measurements across studies.

## Data Availability

The datasets presented in this article are not readily available because of the absence of patient/parent consent for public data sharing. Requests to access the datasets should be directed to a.s.verschuur@alumnus.utwente.nl.
